# Prevalence and Incidence of Diabetes‐Related Peripheral Neuropathy, Peripheral Artery Disease, Foot Ulcers and Lower Extremity Amputations in Ireland; A Systematic Review

**DOI:** 10.1002/jfa2.70147

**Published:** 2026-03-16

**Authors:** Sinead Kavanagh, Jennifer A. Pallin, Ann Sinéad Doherty, Linda M. O'Keeffe, Steven Gilmore, Peter A. Lazzarini, Claire M. Buckley

**Affiliations:** ^1^ School of Public Health University College Cork Cork UK; ^2^ Department of Endocrinology Bantry General Hospital Cork UK; ^3^ Converge: Centre for Chronic Disease and Population Health Research School of Population Health RCSI University of Medicine and Health Sciences Dublin UK; ^4^ Department of General Practice School of Medicine University College Cork Cork UK; ^5^ MRC Integrative Epidemiology Unit at the University of Bristol Bristol UK; ^6^ Population Health Sciences Bristol Medical School University of Bristol Bristol UK; ^7^ School of Public Health and Social Work Queensland University of Technology Brisbane Queensland Australia; ^8^ Allied Health Research Collaborative The Prince Charles Hospital Brisbane Queensland Australia

**Keywords:** diabetic foot, epidemiology, incidence, prevalence

## Abstract

**Introduction:**

Diabetes‐related foot disease is a leading cause of global disease burden, however the prevalence and incidence of diabetes‐related foot disease in Ireland is poorly understood. Up‐to‐date population‐level estimates of the incidence and prevalence are imperative to support appropriate health service planning. This study examined the prevalence and incidence of diabetes‐related foot disease in the Irish population.

**Methods:**

We systematically searched Pubmed, EMBASE and Lenus the Irish Health Research repository, for peer‐reviewed articles published until August 2025. Publications reporting on prevalence and incidence of peripheral neuropathy, peripheral artery disease, foot ulceration or amputation in people with diabetes in Ireland, were eligible for inclusion. The Joanna Briggs Institute Prevalence (JBI) Critical Appraisal Tool was used to assess included studies methodological quality and establish the degree to which bias was addressed in the study's design and analysis. Results were synthesised descriptively according to study characteristics and outcomes.

**Results:**

Three studies met the inclusion criteria (*n* = 145,945), with varying outcome measurement methods. In community‐based diabetes populations, peripheral neuropathy prevalence ranged from 15% to 39% (*n* = 1055) and peripheral artery disease prevalence ranged from 18% to 34% (*n* = 383). For the history of foot ulcers, prevalence was 3.7% (*n* = 563) and annual incidence was 2.6% (*n* = 383). One national population‐based study (*n* = 144,710) reported incidence of amputation increased from 144.2 to 175.7 per 100,000 people with diabetes between 2005 and 2009.

**Conclusion:**

This review found there is a paucity of information on prevalence and incidence of diabetes‐related foot disease in Ireland. However, these findings suggest that prevalence is similar to, if not lower than, global rates of peripheral neuropathy, peripheral artery disease outcomes and amputation incidence outcomes. High heterogeneity in populations and outcomes highlights the need for robust studies and consensus on diabetes‐related foot outcome assessment. Establishing a national diabetes register could strengthen surveillance, identify high‐risk groups and inform cost‐effective public health planning.

**Trial Registration:**

PROSPERO (CRD42023472904)

## 1 Introduction

Diabetes is estimated to affect more than 536 million people worldwide and is rapidly increasing [1]. With this comes increasing diabetes‐related complications such as cardiovascular, kidney, eye and foot disease [1]. Diabetes‐related foot disease (DFD) is defined as disease of the foot in a person with current or previously diagnosed diabetes mellitus that includes one or more of the following: peripheral neuropathy, peripheral artery disease, infection, ulcer(s), neuro‐osteoarthropathy, gangrene or amputation of the foot [2]. Recent estimates suggest DFD affects 199 million people worldwide and causes 1.8% of the global disease burden, making DFD the 13th largest cause of (> 350 causes) of global disease burden [3].

Peripheral neuropathy or peripheral artery disease are well‐known to significantly increase a person's risk of foot ulceration and subsequent amputation [4]. Diabetes‐related foot ulceration (DFUs) and lower extremity amputations (LEAs) are a significant public health concern due to their association with increased hospitalisations, morbidity, mortality, poor quality of life and healthcare costs [5, 6, 7, 8, 9, 10]. To reduce associated morbidity and mortality, international recommendations suggest early identification and referral of those with risk factors for ulceration and amputation to appropriate diabetic foot prevention and management services [11]. However, evidence suggests that many people are not being screened for risk factors and there are insufficient diabetic foot services to manage populations with DFD [12, 13, 14, 15]. One way of overcoming this is by ensuring enough resources (e.g., healthcare professionals, infrastructures and finances) are in place to provide appropriate screening, prevention and management for those at‐risk of foot ulceration and amputation. To ensure this, up‐to‐date population estimates of the prevalence and incidence of these DFD complications are required to allow for appropriate health service planning.

Previous systematic reviews and meta‐analyses indicate that the global prevalence of those with diabetes‐related peripheral neuropathy, peripheral artery disease, foot ulcerations and minor lower extremity amputations are increasing [16, 17]. Conversely, the incidence of major (leg) amputations is decreasing which may be partially explained by increased access to prevention services [18, 19, 20]. Ireland does not have a national diabetes registry and hence, prevalence and incidence of diabetes and associated complications are reliant on estimates from the literature [21]. Reports suggest the incidence of diabetes‐related amputations is increasing in Ireland, however, little is known about the incidence or prevalence of peripheral neuropathy, peripheral artery disease or foot ulceration [22]. Thus, an improved understanding of the epidemiology and burden of DFD in Ireland is needed to inform policymakers on whether increased efforts are needed to support appropriate evidence‐based services for people with diabetes living in Ireland. Therefore, we aimed to systematically review the prevalence and incidence of peripheral neuropathy, peripheral artery disease, foot ulceration and lower extremity amputations in people with diabetes in Ireland.

## 2 Methods

### 2.1 Study Design

This systematic review was designed and performed in line with the Joanna Briggs Institute (JBI) manual for systematic reviews and the Preferred Reporting Items for Systematic Review and Meta‐Analyses (PRISMA) guidelines [23, 24]. See Supporting Information S1: Appendix A for PRISMA checklist. This review was also prospectively registered on PROSPERO database for systematic reviews (CRD42023472904) and the protocol has been published previously [25].

### 2.2 Search Strategy

‘PubMed’ and ‘EMBASE’ databases, along with the Irish based database ‘Lenus’, were systematically searched for studies reporting on the prevalence and/or incidence of the outcomes of interest in the Irish population published in the English language from database inception to January 2024. Backward citation searching of articles selected for full text review was also carried out. The Condition Context Population (CoCoPop) framework was used to inform development of the search strategy [25]. Boolean operators (AND, or and proximity operators were utilised to combine search headings, where appropriate). Full search strategies for each database can be found in Supporting Information S1: Appendix B.

### 2.3 Eligibility Criteria

Inclusion and exclusion criteria are provided in Table 1.

**TABLE 1:** Eligibility criteria.

Population (inclusion criteria)	Included studies were required to report a population of people residing in the republic of Ireland with a diagnosis of diabetes and report at least one of the outcomes of interest. The populations were required to be from a defined geographical catchment area of Ireland (e.g., national population, regional population, inpatient population etc.) due to the requirement for a clearly defined denominator.
Population (exclusion criteria)	Studies were excluded if data relating to an Irish population could not be differentiated from other population groups (e.g., those from the United Kingdom) and where populations could not be differentiated between those with and without a diagnosis of diabetes. Publications from a single centre(s) were excluded, unless general or diabetes populations for the catchment of the selected centre(s) were provided or if data were adjusted or compared to the overall Irish population.
Settings	Studies carried out in community settings (including GP settings, primary care centres etc.) and hospital settings (including inpatient and outpatient) were included.
Study design	All original observational study designs, except for case‐control, case series, case reports and grey literature, were included.
Outcomes	Outcomes of interest were defined by international guidelines and included: peripheral neuropathy, peripheral artery disease, foot ulceration and minor or major lower extremity amputation [2].

### 2.4 Study Screening and Selection

Results from each database were imported into Mendeley and any duplicates removed. Unique records were then exported to Covidence for title and abstract screening. Title and abstract screening, as well as full text assessments, were carried out against the inclusion criteria independently by two reviewers (SK and SG). For any disagreements, a third reviewer (JP or AD) made the final decision.

### 2.5 Data Extraction

Using a standardised data extraction form in Microsoft Word (see Supporting Information S1: Appendix C), data extraction was independently conducted by two reviewers (SK and SG). Extraction was also rechecked by a third and fourth reviewer for accuracy (JP & AD). Disagreements between reviewers were resolved by discussion until consensus was reached.

### 2.6 Quality Assessment and Risk of Bias

The Joanna Briggs Institute Prevalence (JBI) Critical Appraisal Tool was used to assess included studies methodological quality and establish the degree to which bias was addressed in the study's design, conduct and analysis [26, 27]. To assess these parameters, the JBI checklists include nine questions with four answer options: ‘Yes’, ‘No’, ‘Unclear’ and ‘Not applicable’. Assessments were carried out independently by two reviewers (SK and SG) and where disagreements occurred, a third and fourth reviewer were consulted until consensus was reached (JP and AD). Certainty of evidence was not formally assessed.

### 2.7 Synthesis of Results

Eligibility criteria for performing a meta‐analysis were not met and so data was synthesised using a narrative synthesis approach [25]. This allowed for comparison of similarities and disparities between the findings of included studies. This included findings relating to incidence and prevalence of outcomes of interest and information relating to study setting, participant characteristics, measurement of outcomes and methodological quality of included studies.

## 3 Results

**FIGURE 1:**
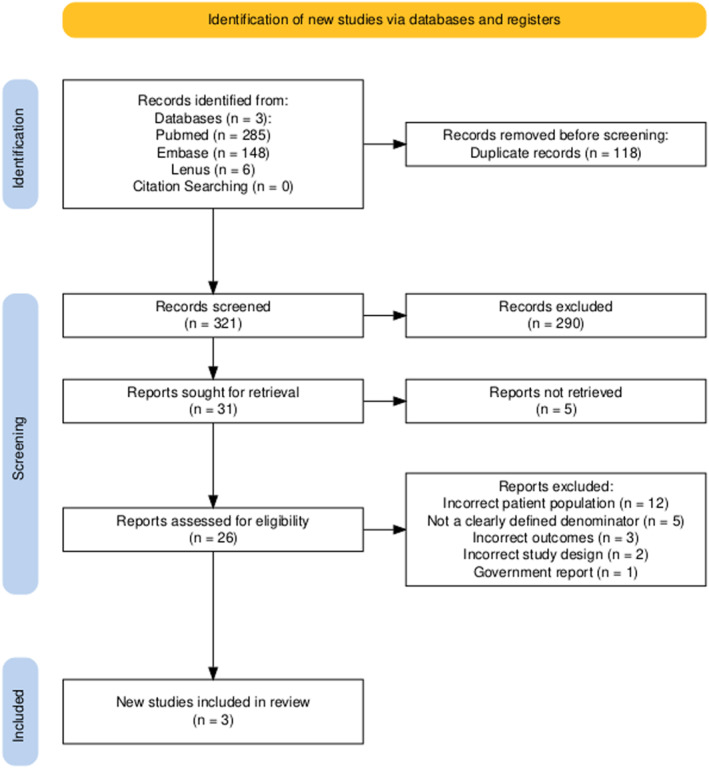
PRISMA flow diagram.

As indicated in the PRISMA flow diagram (Figure 1), 439 publications were identified in the initial search: 285 from PubMed and 148 from EMBASE and six from Lenus. Following duplicate removal, 321 publications were available for title and abstract screening, of which 31 studies were selected for full text screening. Five reports were not retrieved, as these were available only as conference abstracts with no full text. Of the 26 remaining, three studies met the criteria for inclusion (*n* = 145,945).

Table 2 provides an overview of individual study design characteristics, study population characteristics and outcomes of interest reported. Using a retrospective cohort study design, Tracey et al. reported on prevalence of peripheral neuropathy in a community dwelling diabetes population (*n* = 672) [30]. Within this sample, there was a higher proportion of Type 2 diabetes (97.5%) compared to Type 1 diabetes (2.5%). Utilising a national retrospective cohort study using data on hospital discharges for people with diabetes (*n* = 144,710) Buckley et al. reported on incidence of LEAs over a 5‐year period (2005–2009) [28]. Hurley et al. utilised a prospective observational study to examine prevalence of peripheral neuropathy, peripheral artery disease, LEAs and DFUs [29]. They also had a higher proportion of people with Type 2 diabetes (90%) compared to Type 1 diabetes (10%). As outlined in Table 2, outcome measurement methods varied across the included studies with Buckley et al. using hospital discharge data to identify outcomes through International Classification of Diseases 10th Revision (ICD‐10) codes, Hurley et al. conducting clinical examination and medical record review and Tracey et al. using self‐reported data as part of a national study.

**TABLE 2:** A summary of characteristics of the included publications.

Author	Population	Study design	Period	Population size	Diabetes type	Age (years)	Sex	Diabetes duration (years)	Outcomes reported	Outcome measurement
Buckley et al. [28]	National Diabetes Population	Retrospective cohort study	5 years (2005–2009)	*n* = 144,710	Type 1 & 2 (% not provided)	—	—	—	LEAs	Review of hospital discharge data for ICD‐10 codes^a^
Hurley et al. [29]	Community dwelling Diabetes population	Prospective observational study	18 months (2008–2009)	Study baseline *n* = 563 Follow‐up *n* = 383	Type 1 = 10% Type 2 = 90%	64 (± 13.4)	Male = 60% Female = 40%	7.7 (± 8.2)	LEAs DFUs DPN PAD	At study baseline, all participants underwent clinical examination and medical records checked. At follow‐up, patients were reassessed, medical records checked and members of the participants’ medical team questioned.
Tracey et al. [30]	National diabetes population	Retrospective cohort study	2 years (2009–2011)	*n* = 672	Type 1 = 2.5%	50–64 = 43%	Male & female (% not provided)	—	DPN	Self‐reported
					Type 2 = 97.5%	≥ 65 = 57%				

Abbreviations: DFUs, diabetes‐related foot ulcer; DPN, diabetes‐related peripheral neuropathy; LEAs, lower extremity amputations; PAD, peripheral artery disease.

^a^ ICD‐10 Codes: International Classification of Diseases 10th Revision.

### 3.1 Quality Assessment and Risk of Bias

The overall quality of the included studies met the threshold of ‘include’ in the JBI critical appraisal tool for prevalence studies. Sample sizes were adequate, study settings were clearly described and appropriate statistical analyses were undertaken. The studies by Buckley et al. and Hurley et al. met all applicable criteria. In contrast, Tracey et al. did not meet three criteria: two related to the use of self‐reported measures for diabetes‐related foot disease and one concerning the appropriateness of the sampling frame in fully addressing the target population. Despite these limitations, the study was retained and included in the narrative synthesis.

### 3.2 Peripheral Neuropathy

As outlined in Table 3, two studies reported on prevalence of peripheral neuropathy. Both reported various methods of assessments, with one study reporting four different methods. In their study involving community‐based participants (*n* = 563), Hurley and colleagues reported a range of prevalence depending on the measurement tool used. They reported a prevalence of 39% in the total population using the neuropathy disability score. Of this, 7% were found to have mild neuropathy, 16% moderate and 16% severe. Prevalence decreased to 24% when assessed using vibration perception threshold and 25% when assessed using a 10 g monofilament [29]. They also found 10% of the study population had abnormal results across all three sensory tests. In their national retrospective cohort study (*n* = 672), Tracey et al. reported a prevalence of 15% for peripheral neuropathy after asking patients to self‐report presence or absence of peripheral neuropathy [30]. No publication reported peripheral neuropathy incidence.

**TABLE 3:** Summary of the prevalence and incidence findings of the outcomes of interest, with (95% CI)^a^.

		Peripheral neuropathy	Peripheral artery disease	Foot ulceration	Total amputation	Minor amputation	Major amputation
Prevalence:							
Tracey et al. [30]	National Diabetes Population	14.6% (11.4%–18.2%)	—	—	—	—	—
Hurley et al. [29]	Community‐dwelling diabetes population	24%–39%	18%–34%	3.7%	1.6%	—	—
Incidence^b^:							
Buckley et al. [28]	National Diabetes Population	—	—	—	144.2 (123.2–166.9)–175.7 (152.3–200.9) per 100,000	96.2 (78.2–116.3)–127.6 (107.2–150.1) per 100,000	47.9 (37.8–59.5)–48 (37.3–60.4) per 100,000
Hurley et al. [29]	Community‐dwelling diabetes population	—	—	2.6% (1.6%–4.1%)	0.3% (0.1%–1.2%)		—

^a^ Confidence Interval.

^b^ Annual Incidence.

### 3.3 Peripheral Artery Disease

Following clinical examination, Hurley et al. (*n* = 383) reported a prevalence 18% based on two or more pedal pulses being absent. This increased to 34% when assessed using vascular symptoms and absence of triphasic pulses (Intermittent claudication; 10%, Night Pain: 17% and Rest pain: 7%). However, it is not reported whether those who had vascular symptoms also had absent pedal pulses [29].

### 3.4 Foot Ulceration

No study reported active foot ulcer prevalence. One publication, Hurley et al. reported a history of ulceration prevalence of 3.7% (*n* = 563). This was based on participants self‐reporting a history of ulceration, confirmed by checking medical records. A subset of this population was followed for 18 months (*n* = 383), reporting an annual incidence of a new foot ulcer of 2.6% (95% CI, 1.6%–4.1%) [29].

### 3.5 Lower Extremity Amputation

Hurley et al. reported a prevalence of 1.6% of LEA history based on a medical record review of 563 individuals. The same study followed 383 participants for 18 months, reporting an annual incidence of 0.3% (95% CI, 0.1%–1.2%) [29]. For total amputation, a national population‐based study (*n* = 144,710) reported an incidence of 144.2 per 100,000 people with diabetes in 2005, increasing to 175.7 per 100,000 people with diabetes in 2009. When separated by amputation type, the incidence of minor amputations was 92.6 per 100,000 people with diabetes in 2005 and 127.6 per 100,000 people with diabetes in 2009. The incidence of major amputations was 47.9–48.0 per 100,000 people with diabetes in both 2005 and 2009 [28]. ICD‐10 codes were used to ascertain amputation diagnosis and type.

## 4 Discussion

This systematic review identified three studies reporting on incidence and prevalence of peripheral neuropathy, peripheral artery disease, DFUs or LEAs in people with diabetes in the Republic of Ireland. Despite conducting an extensive search across multiple databases, only three studies met our inclusion criteria. This reflects the scarcity of population‐based research on DFD incidence and prevalence in Ireland. Most excluded studies lacked a clearly defined denominator or did not report the relevant outcomes of interest. Although publication bias cannot be entirely ruled out, the limited number of eligible studies is more plausibly explained by the lack of research in this area. Further, there was substantial heterogeneity in the methodology used to measure and report the four outcomes.

This review found the prevalence of peripheral neuropathy ranged from 15% to 39% in community dwelling populations with diabetes. However, methods of ascertainment and population characteristics differed between studies, making it difficult to compare and estimate the prevalence of neuropathy in the Irish population. For example, Hurley et al., reported a prevalence of 23%–39% depending on method of assessment, with the neuropathy symptoms score indicating higher levels of neuropathy and the vibration perception threshold indicating lowest level of neuropathy. Although the lower rate is comparable to observational studies involving community‐based populations [31, 32], the higher rate is comparable to the pooled global estimate of 34% and provides the most reliable estimate for informing epidemiological results in this paper [33]. In their study of adults above the age of 50, which reported a prevalence of 15%, Tracey et al. used data from a broad longitudinal study of ageing; participants were asked to self‐report if a healthcare provider has ‘ever told them they have a lack of feeling and tinging legs and feet due to nerve damage [30]. This method for identifying neuropathy has not been validated and may have led to an inaccurate estimation of prevalence, as up to 50% of people with peripheral neuropathy may be asymptomatic [34] and people with diabetes are often unaware of their foot risk status even when screened [35].

This review suggests the prevalence of peripheral artery disease varies depending on the measurement tool used. As of 2023, there is international agreement on what assessment tool to screen for peripheral artery disease in clinical practice [36]. In the included study in this review, this was not available at the time of publication to enable effective comparison between studies. The framework requires Ankle‐brachial pressure; Ankle systolic pressure and a transcutaneous oxygen pressure (TcPO_2_) or Toe Pressure. The observational study identified reported a prevalence of 18%–34% depending on method of diagnosis for peripheral artery disease. The prevalence of 18% was based on an absence of two or more pedal pulses. Using the same diagnostic criteria, results from a community‐based study in the United Kingdom reported a prevalence of 21% [37]. A previous review of incidence and prevalence of DFD in Australian populations when using the WIfi framework, reported a prevalence of 10%–29% in community‐based populations and 35% in inpatient populations [16].

We identified one study examining incidence and prevalence of DFUs, reporting an annual incidence of 2.6% in a community‐based cohort. However, they did not report whether these were first ulcerations or recurrent. We found that although no study reported the active prevalence of DFUs, one publication provided insights into the history of ulceration prevalence and incidence in a community‐dwelling population. Hurley et al. (2013) reported a prevalence of 3.7% for individuals with a self‐reported history of ulceration, which was subsequently confirmed by medical record review. Internationally, incidence of active DFUs ranges from 0.15% to 8.8% with higher incidences seen in hospital settings [37, 38, 39]. In the United Kingdom, one study involving a community‐based population cohort of similar age and diabetes duration reported an annual incidence rate of 2.2% [37]. Global prevalence of DFUs is estimated to be 6.3% and in Europe it is estimated to be 5.5% which is slightly higher than the prevalence in the study identified within this review [17]. However, the Global and European studies used pooled data from hospital clinics, public health clinics and population‐based studies [17]. Using global estimates and based on the most recent estimation of the Irish Diabetes population of 327,927, this suggests 11,897 patients are living with diabetes‐related foot disease in Ireland [40]. However, in the absence of a national diabetes register and thus recording of these outcomes at a national level DFU burden is not clear at present.

We identified one study providing insight into changes over time in amputation rates in people with diabetes in Ireland. Internationally, there are variations in incidence between and within countries [41, 42, 43, 44]. However, reported global incidence of total amputation ranges from 78 to 704 per 100,000 person‐years [41]. A more recent study reported the median (range) initial rate was 3.1 total amputation admissions per 1000 person‐years with diabetes [41]. Our review found total amputation rates increased from 144.2 per 100,000 people with diabetes in 2005 to 175.7 per 100,000 people with diabetes in 2009. Although this study found incidence was increasing, global estimates suggest rates are decreasing [45, 46]. Furthermore, reductions are being driven by declines in major LEAs rather than declines in minor LEAs whereas this review found major amputations rates in Ireland are increasing. It is well recognised that minor amputations are an attempt at limb salvage whereas major amputation characterises failed limb salvage and is often required to save a life [47]. In addition, amputation rates decrease where individuals have access to organised diabetic foot care involving screening, timely referral to foot care specialists and timely implementation of treatment and management strategies [20, 48, 49, 50, 51]. Results from observational studies suggest that organised diabetes‐related foot care has not been implemented in Ireland, potentially accounting for this increase in LEAs [13, 15]. However, updated figures on amputations rates in people with diabetes in Ireland are needed to examine the impact of national policy in Ireland.

### 4.1 Strengths and Limitations

This study has several strengths. To the best of our knowledge, this is the first study to systematically review the literature for multiple DFD complications in Ireland and provide evidence on the burden in Ireland. Secondly, the requirement of a clearly defined denominator in included studies is also a strength. Publications from a single centre were excluded, unless general or diabetes‐population information for the selected centre was provided or if data was adjusted/compared to the overall Irish population demographics. This criterion allowed us to improve the external validity of our results.

However, a limitation is that, although we searched databases using a robust search strategy, we did not include grey literature meaning we may have missed out on some data. A further limitation is that this was a narrative review. Although we had planned on completing a meta‐analysis, this was not possible with the subsequent outcomes. Third, all studies used varying outcome measurement methods, which significantly limited the ability to compare findings across studies. This lack of standardisation in diagnostic criteria, assessment tools and reporting frameworks is a critical barrier to understanding the true burden of diabetes‐related foot complications in Ireland. The variation in outcome definitions and ascertainment methods compromises the internal validity of individual studies and undermines efforts to draw broader conclusions about incidence and prevalence trends nationally.

### 4.2 Recommendations

Given that no study reported the active prevalence of DFUs, it is clear that future research should focus on capturing data on both the prevalence and incidence of active foot ulcers. More robust and comprehensive longitudinal studies with standardised methods for foot ulcer identification would provide a clearer picture of the burden of foot ulcers in various populations. Further studies of nationally representative populations using valid outcomes measures are needed to provide insight into DFD complications in Ireland. Establishing a national diabetes register or using routinely collected data, such as hospital episodes, primary care records and prescriptions could enhance DFD surveillance. These systems would enable monitoring of prevalence and incidence, identify high‐risk groups and inform public health planning in a cost‐effective way.

## 5 Conclusion

This review highlights a paucity of information on the prevalence and incidence of DFD in Ireland. The available data identified within this review suggest that the prevalence of conditions such as peripheral neuropathy, peripheral artery disease and outcomes related to amputation in Irish populations may be similar to or potentially lower than, global rates. However, the lack of comprehensive and consistent data makes it difficult to draw definitive conclusions about the burden of these conditions in Ireland. The findings underscore the need for more robust and ongoing studies to better capture and compare the prevalence and incidence of DFD in the Irish population.

## Author Contributions

**Sinead Kavanagh:** conceptualization, methodology, writing – original draft preparation, writing – review and editing. **Jennifer A. Pallin:** conceptualization, methodology, supervision, writing – original draft preparation, writing – review and editing. **Ann Sinéad Doherty:** methodology, supervision, writing – review and editing. **Linda M. O'Keeffe:** methodology, writing – review and editing. **Steven Gilmore:** methodology, writing – original draft preparation, writing – review and editing. **Peter A. Lazzarini:** methodology, writing – review and editing. **Claire M. Buckley:** conceptualization, methodology, supervision, writing – review and editing.

## Funding

The authors have nothing to report.

## Conflicts of Interest

The authors declare no conflicts of interest.

## Supporting information


Supporting Information S1

## Data Availability

The data that supports the findings of this study are available in the supplementary material of this article.
